# Potent NKT cell ligands overcome SARS-CoV-2 immune evasion to mitigate viral pathogenesis in mouse models

**DOI:** 10.1371/journal.ppat.1011240

**Published:** 2023-03-24

**Authors:** Hongjia Lu, Zhewei Liu, Xiangxue Deng, Siyang Chen, Ruiting Zhou, Rongqi Zhao, Ramya Parandaman, Amarjot Thind, Jill Henley, Lei Tian, Jianhua Yu, Lucio Comai, Pinghui Feng, Weiming Yuan

**Affiliations:** 1 Department of Molecular Microbiology and Immunology, Keck School of Medicine, University of Southern California, Los Angeles, California, United States of America; 2 Graduate Programs in Biomedical and Biological Sciences, Keck School of Medicine, University of Southern California, Los Angeles, California, United States of America; 3 The Hastings and Wright Laboratories, Keck School of Medicine, University Southern California, California, United States of America; 4 Department of Hematology and Hematopoietic Cell Transplantation, City of Hope National Medical Center, Los Angeles, California, United States of America; 5 Section of Infection and Immunity, Herman Ostrow School of Dentistry, University of Southern California, Los Angeles, California, United States of America; University of Rochester Medical Center, UNITED STATES

## Abstract

One of the major pathogenesis mechanisms of SARS-CoV-2 is its potent suppression of innate immunity, including blocking the production of type I interferons. However, it is unknown whether and how the virus interacts with different innate-like T cells, including NKT, MAIT and γδ T cells. Here we reported that upon SARS-CoV-2 infection, invariant NKT (iNKT) cells rapidly trafficked to infected lung tissues from the periphery. We discovered that the envelope (E) protein of SARS-CoV-2 efficiently down-regulated the cell surface expression of the antigen-presenting molecule, CD1d, to suppress the function of iNKT cells. E protein is a small membrane protein and a viroporin that plays important roles in virion packaging and envelopment during viral morphogenesis. We showed that the transmembrane domain of E protein was responsible for suppressing CD1d expression by specifically reducing the level of mature, post-ER forms of CD1d, suggesting that it suppressed the trafficking of CD1d proteins and led to their degradation. Point mutations demonstrated that the putative ion channel function was required for suppression of CD1d expression and inhibition of the ion channel function using small chemicals rescued the CD1d expression. Importantly, we discovered that among seven human coronaviruses, only E proteins from highly pathogenic coronaviruses including SARS-CoV-2, SARS-CoV and MERS suppressed CD1d expression, whereas the E proteins of human common cold coronaviruses, HCoV-OC43, HCoV-229E, HCoV-NL63 and HCoV-HKU1, did not. These results suggested that E protein-mediated evasion of NKT cell function was likely an important pathogenesis factor, enhancing the virulence of these highly pathogenic coronaviruses. Remarkably, activation of iNKT cells with their glycolipid ligands, both prophylactically and therapeutically, overcame the putative viral immune evasion, significantly mitigated viral pathogenesis and improved host survival in mice. Our results suggested a novel NKT cell-based anti-SARS-CoV-2 therapeutic approach.

## Author summary

The ongoing COVID-19 pandemic is the most severe public health crisis in recent history. Even with efficacious vaccines, it is imperative to better understand the mechanism of viral pathogenesis particularly how SARS-CoV-2 suppresses human innate immunity in order to develop more powerful and effective treatments, not only for the current pandemic, but also for the potential re-emergence of new variants. Here we reported the first in-depth characterization of SARS-CoV-2 interaction with the important arm of innate immunity, CD1d-restricted natural killer T (NKT) cells. We discovered NKT cells were rapidly recruited to lungs post infection. The virus employed its envelope protein to suppress the expression of the key antigen-presenting molecule, CD1d, and inhibited the activation of innate NKT cells. Remarkably, we discovered that this immune evasion was unique to highly pathogenic coronaviruses and absent from common cold coronaviruses, suggesting that this new immune evasion mechanism was an important virulence factor. More importantly, we demonstrated that activation of invariant NKT cells significantly overcame the viral immune evasion and improved the host survival.

## Introduction

The ongoing COVID-19 pandemic is the most severe public health crisis in over a hundred years. With extremely high contagiousness and potent pathogenicity, the etiological SARS-CoV-2 virus caused unprecedented mortality and morbidity worldwide. Even with efficacious vaccines, it is imperative to better understand the peculiar pattern of pathogenesis and develop powerful and effective treatment strategies not only for this lingering pandemic but also for future potential re-emergence of new variants. Accordingly, it is critical to delineate the mechanism of viral pathogenesis as well as the roles of major arms of the human immune system during SARS-CoV-2 infection. NKT cells are a potent group of innate-like T cells playing effective immune-regulatory function during infectious diseases.

As a critical component of innate immunity, CD1d-restricted Natural Killer T (NKT) cells are an unconventional subset of T cells co-expressing T-cell receptor (TCR) and typical surface receptors for NK cells [1]. Over the last two decades, studies have shown NKT cells play potent immunoregulatory functions in diverse immune responses, particularly in fighting infectious diseases and tumors. The major population of NKT cells expresses a single TCRα chain, Vα24Jα18 in human and Vα14Jα18 in mice, and is often called invariant NKT (iNKT) cells. Innate-like NKT cells are among the first responders in the periphery during immune responses and are typically activated within hours. Upon activation, they rapidly produce copious amount of cytokines, both Th1 and Th2 types, and play powerful immunomodulatory function for ensuing adaptive immune responses [2]. Different from MHC class I and II, which present peptide ligands to conventional T cells, CD1d molecules present antigenic phospholipids or glycolipids to NKT cells [3,4]. While NKT cells have demonstrated their particularly strong anti-tumor function, it is believed that innate-like T cells are first evolved for their anti-infection function [5]. This hypothesis is supported by numerous reports showing their antiviral and anti-bacterial functions [6,7]. Many viruses, including HIV [8–10], HSV-1 [11,12], KSHV [13] and LCMV [14] use different mechanisms to suppress CD1d and NKT cell function. A common tactic adopted by viruses to evade T cell function is to down-regulate the antigen-presenting molecules [15,16]. In nearly all viruses studied so far, they achieve the blocking of NKT cell function via down-regulation of CD1d expression [11,13,17].

SARS-CoV-2 virus belongs to the β-coronavirus family, which also includes SARS and MERS viruses [18]. With a large genome of approximately 30 Kb, the virus encodes 4 structure proteins, Spike (S), Membrane (M), Envelope (E) and Nucleocapsid (N) protein and 16 nonstructural proteins (Nsp 1–16) processed from two overlapping large ORFs (ORF1a and ORF1b). In addition, the virus encodes several associate proteins (ORF3a, 3b, 6, 7a, 7b, 8, 9a, 9b and 10) in the 3’ end of its genome [18]. Many elegant studies have reported potent immune evasion and suppression mechanisms by SARS coronavirus [19–21]. One prominent strategy employed by this prototype virus is its efficient blocking of type I interferon production [20,21]. In human innate immune systems, there are also large numbers of innate lymphocytes playing critical roles in antiviral immunity [22,23]. Genetic studies have demonstrated their potent antiviral function towards diverse virus groups [24,25]. Among these innate lymphocytes, two groups of innate T cells are prominent members, the CD1d-restricted NKT cells and the MR1-restricted MAIT cells [22,26]. Recent literature has documented their emerging roles against viral infections in lungs [27]. While MAIT cells are more regulated by the immune experience of the host, the abundance and composition of NKT cells are controlled by both genetics and host immune experience [28]. One salient feature of NKT cell function is their potent regulatory function in the entire immune responses, from the onset of infection to disease progression and to the recovery/tissue repair stages due to their unique capacity of secreting both Th1 and Th2 types of cytokines [29]. NKT cells play potent anti-infection functions in lungs against the bacterial pathogens, including the common pneumonia-causing bacterial pathogen, *Streptococcus pneumonia* [30,31], as well as the common viral pathogen, influenza [32]. For an aggressive pathogen like SARS-CoV-2 virus, it is possible that this virus has also evolved mechanisms to suppress the function of innate lymphocytes including NKT cells.

Many clinical reports have documented a distinct role of NKT cells in anti-COVID 19 immune responses [33–36]. When comparing lymphocyte numbers in mild patients versus those in healthy controls, there was no significant change in levels of NKT or NK, CD4 and CD8 T cells in peripheral blood. However, in severe patients, all innate lymphocytes showed reduced counts in the blood when compared to that of healthy controls [33]. The general decrease in NKT cells as well as other innate-like T cells over time in severe stages of disease is consistent with the idea that these T cells play a more important antiviral function in the early phases. Consistently, an immediately increased expression of CD69 in iNKT cells was observed at the time of initial infection [34]. In severe patients, while the frequency of circulating iNKT cells was lowered, a hyperactivated phenotype of iNKT cells was detected in airway/lung tissue including raised expression of IL-6 and IL-17A. This is most likely due to the functional alteration of iNKT cells induced by elevated levels of plasma IL-18 [34]. We are interested in understanding whether and how CD1d-restricted NKT cells play a significant role in anti-SARS-CoV-2 immune responses and whether the virus has evolved specific evasion mechanisms to antagonize the antiviral function of NKT cells. We are also interested to investigate whether pharmacological activation of NKT cells can mitigate the diseases caused by SARS-CoV-2 and improve the host survival.

## Results

### 1. Upon infection, SARS-CoV-2 rapidly reduces iNKT cell numbers in periphery and suppresses CD1d expression

**Fig 1:**
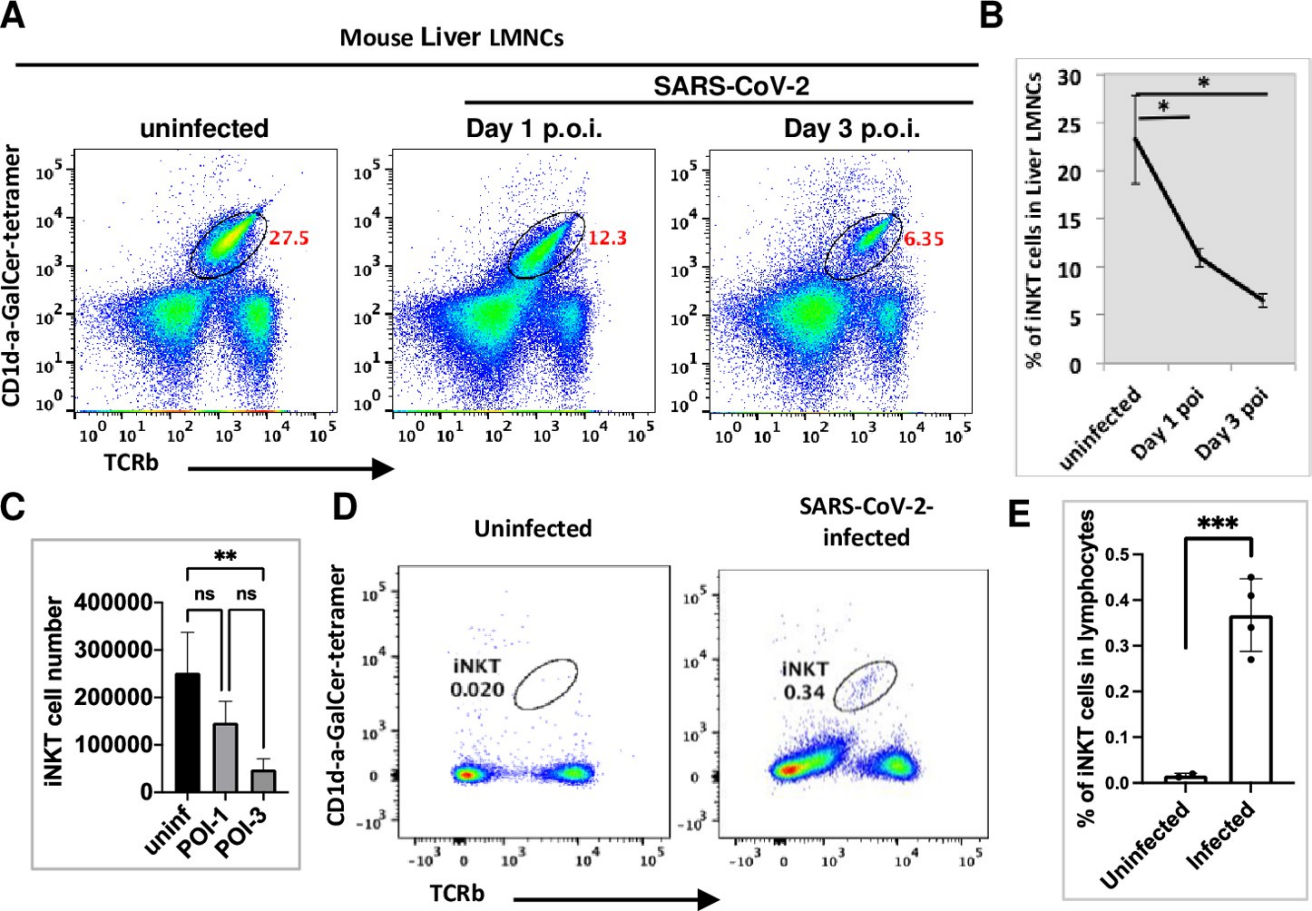
SARS-CoV-2 infection reduces iNKT cell abundance in periphery but increases iNKT cell numbers in the lungs of infected mice. (A). Upon infection, SARS-CoV-2 rapidly reduced iNKT cell numbers in mouse livers. K18-hACE2-Tg mice (n = 3) were infected with SARS-CoV-2 (Isolate USA-WA1/2020) at 1X10e4 pfu/mouse intranasally. Liver mononuclear cells (LMNCs) were stained with α-GalCer-loaded mouse CD1d tetramer for iNKT cells at 1 or 3 days post-infection. The percentage and total numbers of iNKT cells in LMNCs were plotted over the infection course (B, C). (D, E). Single cells were prepared from SARS-CoV-2-infected mouse lungs and stained for iNKT cells with α-GalCer-loaded CD1d tetramers. Percentage and total numbers of iNKT cells in LMNCs (B, C) and lung lymphocytes (E) were plotted as the mean ± s.d. P values were calculated with two-side unpaired Student’s test. *, **, ***: p<0.05, p<0.01, p<0.001. ns: not significant.

As an initial step to investigate the interaction of virus and NKT cells, we examined the dynamics of the NKT cells in infected mice. Groups of K18-hACE2Tg mice were either uninfected or infected with 1X10e4 pfu/mouse SARS-CoV-2 (WT, Isolate USA-WA1/2020, BEI) and euthanized at Day 1 or Day 3 post infection. Mouse liver mononuclear cells (LMNCs) were prepared and co-stained with anti-TCRβ and CD1d-α-GalCer-tetramer (NIH Tetramer Core Facility), the canonical staining to define iNKT cells [37]. The uninfected hACE2Tg mice had iNKT cell abundance similar to wild-type C57BL6 mice, with 20–30% LMNCs being iNKT cells (Fig 1A). Remarkably, the percentage and total numbers of liver iNKT cells in lymphocytes underwent a rapid, significant and progressive decline in first days post infection, approximately 12% and 6% of total lymphocytes on Day 1 and 3, respectively (Fig 1B and 1C), consistent with clinical reports of a sharp decrease of peripheral NKT cells in severe COVID-19 patients [33,34]. One proposed mechanism for the decreased level of NKT cells was the rapid homing and infiltration of NKT cells into infected lung tissues.

To investigate whether NKT cells are indeed recruited to infected lungs, we examined the NKT cell abundance during SARS-CoV-2 infection. While barely any iNKT cells could be detected in the lungs of uninfected mice (Fig 1D, left), a substantial amount of iNKT cells (approximately 0.3% of total lymphocytes) were detected at three days post infection (Fig 1D and 1E). These results suggested that iNKT cells were recruited to mouse lungs shortly after infection, presumably exerting their antiviral function. It is currently unknown whether the iNKT cells detected in the mouse lungs were from the blood stream or from other peripheral organs including the liver. The trafficking of NKT cells in vivo is mostly mediated by chemotaxis and dependent on the expression of chemokine receptors on NKT cells [38,39]. The local inflammation and tissue damage release chemokines, which can lead to recruitment of NKT cells to infection sites. Such recruitment may not be directly dependent on the CD1d expression on professional or non-professional antigen-presenting cells. We were not too surprised by the accumulation of NKT cells in the lungs as previous studies have documented iNKT cell recruitment in mouse lungs infected by influenza viruses [40,41]. It is currently unknown whether the iNKT cells we detected in mouse lungs post infection were indeed recruited from the livers. There have been reports suggesting NKT cells can be recruited from peripheral blood to infection sites [38] and can exit liver tissues in injury and disease conditions [42]. It will be very interesting to investigate whether the liver iNKT cells can be recruited to lung tissues post SARS-CoV-2 infection.

**Fig 2:**
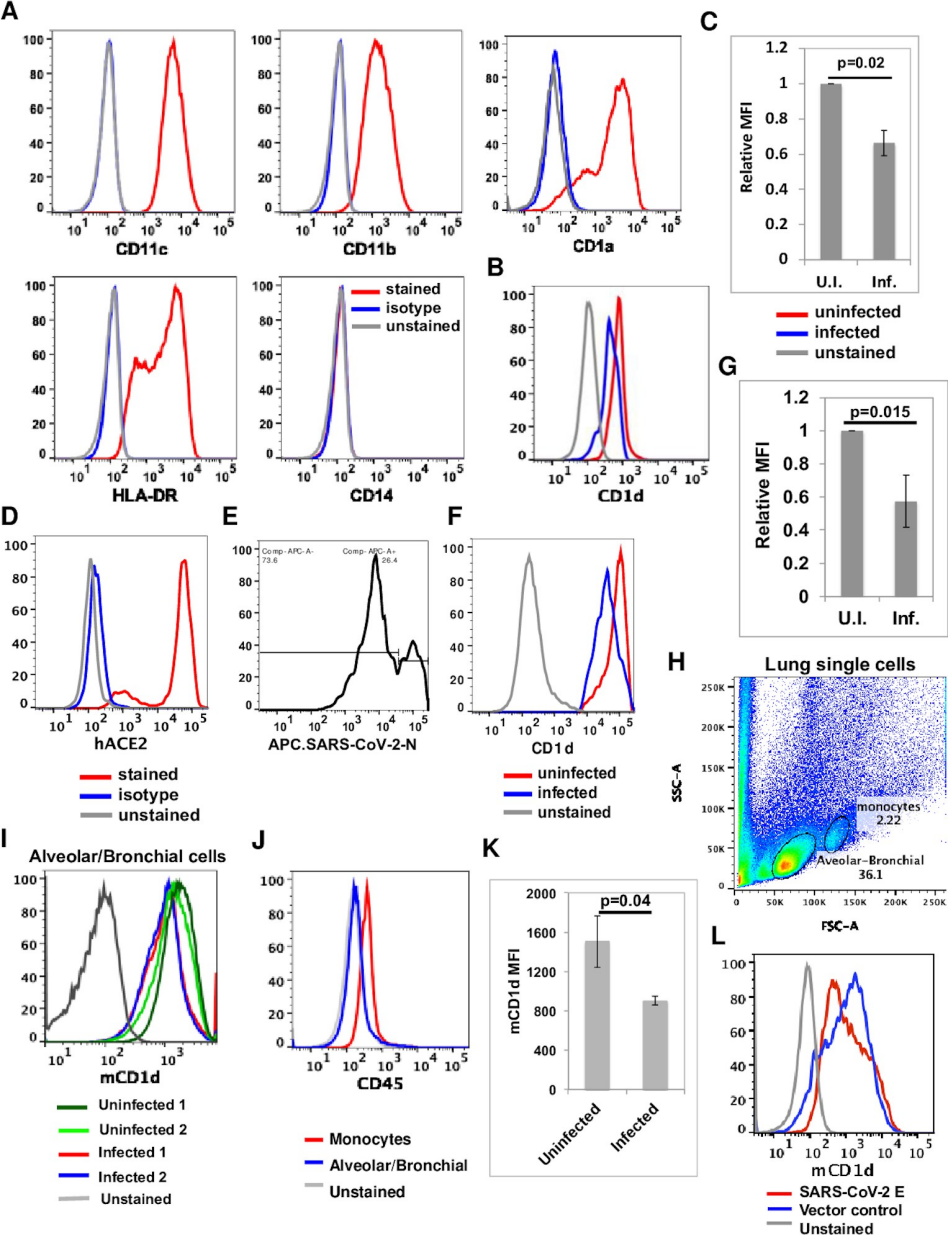
SARS-CoV-2 infection downregulates CD1d in antigen-presenting cells. (A-C). Human peripheral blood monocytes were purified from a healthy donor, derived into dendritic cells, stained with surface markers (A) and infected with SARS-CoV-2 (Isolate USA-WA1/2020) at m.o.i. of 10. At 24 hours post infection, dendritic cells were fixed by paraformaldehyde and stained with anti-CD1d monoclonal antibody CD1d.51.1 then permeabilized and stained with antibodies against viral nucleocapsid (N) protein. Cell surface CD1d expression was compared between infected (N-positive) and uninfected (N-negative) dendritic cells (B, C). (D-G). Sorted HeLa.CD1d.hACE2 cells were stained for expression of human ACE2 protein (D), infected with SARS-CoV-2 (Isolate USA-WA1/2020) at a m.o.i. of 1, fixed and stained for cell surface CD1d and then intracellular N protein as in (E). Cell surface CD1d expression was compared in infected (N protein-positive) and uninfected (N protein-negative) cells (F). Relative CD1d MFI is calculated as CD1d MFI in infected cells divided by CD1d MFI in uninfected cells. Infection experiments were repeated total three times and representative FACS plots are presented. (C.G). Relative CD1d MFI values were calculated and plotted as the mean ± s.d. P values were calculated with two-side unpaired Student’s test. (H-L). SARS-CoV-2 infection downregulates the expression of mouse CD1d. (H-K). hACE2-Tg mice (n = 2) were infected with SARS-CoV-2 (Isolate USA-WA1/2020, 1X10e4 pfu per mouse). Single cells were prepared from mouse lungs three days post infection and blocked with anti-CD16/CD32 antibodies before being stained for anti-CD45 (J) and anti-mouse CD1d monoclonal antibodies (I). Mean fluorescence intensity (MFI) of mouse CD1d staining in alveolar/bronchial epithelial cells from uninfected and infected mice were plotted and compared. Statistical analyses were performed using two-side unpaired Student’s t-test (K). (L). 293T cells were transiently transfected with pLPCX.CD1d and pTracer in the presence of SARS-CoV-2 E protein-expressing pLVX plasmid or pLVX plasmid only (vector control). Transfected cells were stained with anti-mouse CD1d monoclonal antibodies. Mouse CD1d stainings in GFP-positive cells from two transfections were plotted and compared.

To investigate whether SARS-CoV-2 has evolved mechanisms to antagonize the antiviral function of NKT cells, we first investigated whether the virus suppresses the function of the key antigen-presenting molecule, CD1d for NKT cells. We infected human peripheral blood monocytes (PBMC)-derived dendritic cells (DCs, CD11c+CD11b+CD1a+HLA-DR+CD14- and Fig 2A) with SARS-CoV-2 (Isolate USA-WA1/2020, BEI). Despite the low expression of CD1d in DCs, we detected a substantial and reproducible decrease of CD1d expression in infected DCs 24 hours post infection (Fig 2B and 2C). Since the infection of human DCs is not efficient and most likely non-productive [43–47], we then used stable CD1d-expressing cell line for our study of CD1d downregulation and identification of viral gene(s) responsible for the evasion of CD1d function.

Most NKT cells typically reside in local tissues in the periphery [1] and CD1d expression by local epithelial cells play important roles in activating local NKT cells [48,49]. The primary target cells of SARS-CoV-2 are mostly lung alveolar epithelial cells [50]. To investigate whether SARS-CoV-2 infection downregulates CD1d in epithelial cells, we employed epithelial-like HeLa cells stably expressing CD1d and human ACE2 (hACE2) receptor. The expression of hACE2 was verified by flow cytometry (Fig 2D). HeLa.CD1d.hACE2 cells were infected by SARS-CoV-2 (Isolate USA-WA1/2020) at m.o.i. of 1. Twenty-four hours post SARS-CoV-2 infection, cells were first stained for cell surface CD1d with anti-CD1d monoclonal antibody CD1d.51.1 followed by permeabilization and intracellular staining with anti-N protein antibodies. The infection efficiency was significantly higher than that in dendritic cells, with 26.4% cells expressing N protein (Fig 2E). CD1d expression in infected HeLa cells was reduced by approximately 40% (Fig 2F and 2G), suggesting that CD1d was indeed substantially downregulated upon SARS-CoV-2 infection.

To examine whether SARS-CoV-2 downregulates CD1d in our mouse models, we prepared single cells from mouse lungs three days post infection and stained for mouse CD1d expression (Fig 2H–2K). Most of the single cells were non-hematopoietic alveolar/bronchial cells (CD45-negative) with a smaller population of monocytes (CD45-low) (Fig 2H and 2J). Mouse lung single cells were blocked with FcR antibodies (anti-Mouse CD16/CD32, Invitrogen) and stained with an anti-mCD1d mAb (1B1, BD Pharmingen). In comparison to the cells prepared from uninfected mice, the cell surface CD1d expression is indeed lower after SARS-CoV-2 infection by approximately 40% (Fig 2I and 2K), suggesting the viral immune evasion mechanism of CD1d downregulation is operating during SARS-CoV-2 infections in the mouse model.

### 2. SARS-CoV-2 envelope (E) protein is responsible for downregulating CD1d expression and suppressing NKT cell function

**Fig 3:**
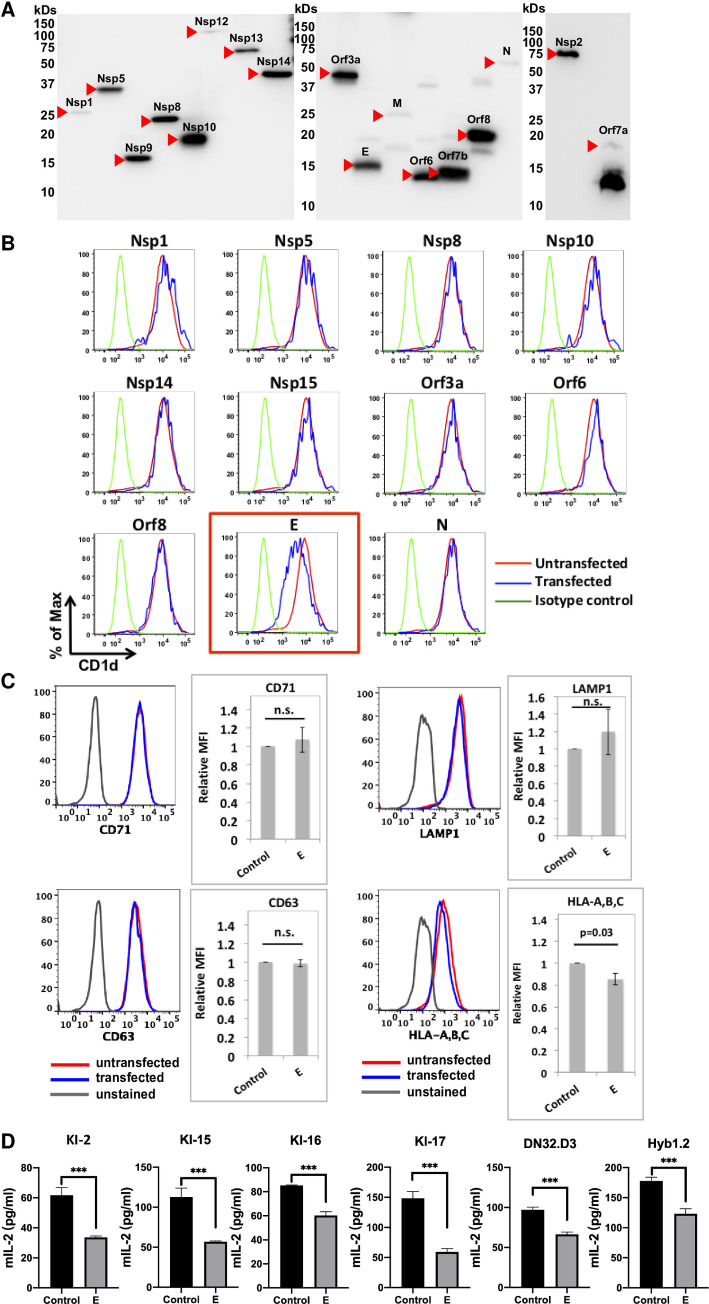
SARS-CoV-2 employs its envelope (E) protein to downregulate CD1d surface expression and suppresses the activation of NKT cells. (A). Verification of viral gene expression in a SARS-CoV-2 expression library. Individual library clones were expressed in 293T cells and cell lysates were western blotted for Strep tag. (B). Identification of SARS-CoV-2 E protein as the major viral gene downregulating CD1d. 293T.CD1d cells were transiently co-transfected with pLVX constructs expressing individual SARS-CoV-2 genes and pTracer vector expressing GFP. Cell surface CD1d expression levels in untransfected (GFP-negative) and transfected (GFP-positive) cells were compared by anti-CD1d (CD1d.51.1) antibody staining and flow cytometry 48 hours post transfection. GFP expression was used as a surrogate marker for transfection. (C). SARS-CoV-2 E protein specifically downregulates CD1d from cell surface. 293T.CD1d cells were transiently co-transfected with pLVX constructs expressing SARS-CoV-2 E protein and pTracer vector expressing GFP. Expression levels of cell surface markers in untransfected (GFP-negative) and transfected (GFP-positive) cells were compared by stainings with anti-CD71, Lamp1, CD63 and MHC class I/HLA-A,B,C antibodies and flow cytometry 48 hours post transfection. GFP expression is used as a surrogate marker for transfection. (D). The CD1d downregulation led to suppressed NKT cell function. Transfected 293T.CD1d cells expressing SARS-CoV-2 E protein were used as antigen-presenting cells, loaded with α-GalCer (100 ng/ml) and co-cultured with different iNKT cell hybridomas for 48 hours. Culture media were collected and subjected to mouse IL-2 ELISA to measure iNKT cell activation.

To identify viral gene(s) responsible for downregulating CD1d expression, we employed an expression library of SARS-CoV-2 [51] to express individual genes and screen for viral gene(s) downregulating CD1d, using 293T.CD1d cells because they are more readily transfected than HeLa.CD1d.ACE2. Expression of SARS-CoV-2 gene products was confirmed by western blotting as Strep-tagged proteins ([51] and Fig 3A). In this near-complete screening of SARS-CoV-2 genes, only one gene product, the envelope (E) protein, efficiently downregulated CD1d expression (Fig 3B). This downregulation was highly specific as we did not detect downregulation of several other cell surface markers including transferrin receptor/CD71, LAMP1 or CD63. The cell surface expression of MHC class I molecules was only slightly decreased (Fig 3C). To investigate whether SARS-CoV-2 E protein is responsible for the CD1d downregulation in mouse infection (Fig 2I and 2K), we transiently transfected 293T cells with plasmids expressing mouse CD1d in the presence or absence of E protein expression. Co-expression of SARS-CoV-2 E protein clearly downregulated cell surface expression of mouse CD1d (Fig 2L), suggesting that SARS-CoV-2 E protein can downregulate mouse CD1d expression during SARS-CoV-2 infection of mice.

To examine whether the E protein-mediated CD1d downregulation suppresses NKT cell function, antigen-presentation assays were performed with six independent iNKT cell hybridoma clones with 293T.CD1d (either untransfected or expressing E protein) as antigen-presenting cells and the prototype glycolipid iNKT ligand, α-galactosylceramide (α-GalCer) as the antigens. Suppression of CD1d expression led to significant inhibition of iNKT cell activation (measured by IL-2 secretion) for all six hybridoma clones with different TCRs (Fig 3D), strongly suggesting the downregulation of CD1d expression leads to suppressing of iNKT cell function.

### 3. SARS-CoV-2 E protein interacts with CD1d molecules and specifically degrades the mature form of CD1d

**Fig 4:**
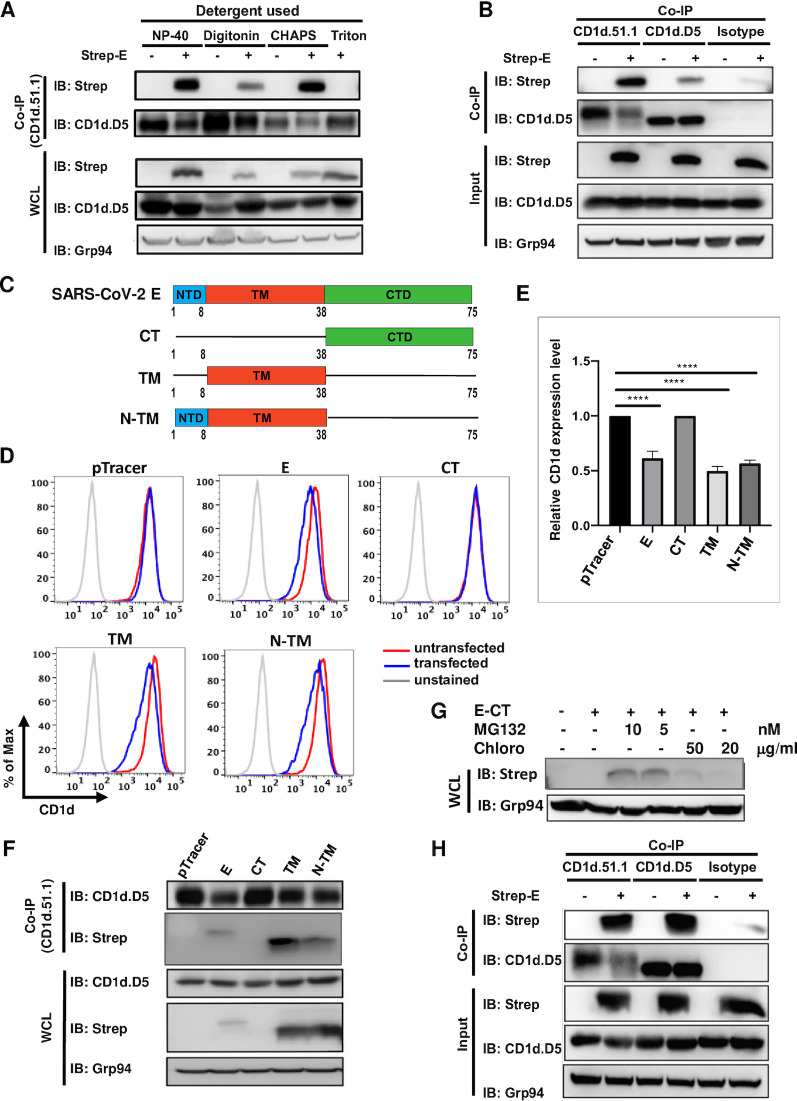
SARS-CoV-2 E protein specifically interacts with mature form of CD1d and leads to its degradation. (A). 293T.CD1d cells were transfected to express Strep-tagged E protein, lysed in 1% indicated detergents and subjected to immunoprecipitation with a mature form-specific anti-CD1d antibody, CD1d.51.1. Immunoprecipitated proteins were western blotted for CD1d and E protein with anti-CD1d antibody (CD1d.D5) or anti-Strep antibody, respectively. Whole cell lysate (WCL) was also blotted by anti-CD1d (CD1d.D5), anti-Strep and anti-Grp94 antibodies. (B). 293T.CD1d cells were transfected to express Strep-tagged E protein and lysed in 1% CHAPS. The cell lysates were immunoprecipitated with either anti-CD1d antibodies (CD1d.51.1 or CD1d.D5, specific for mature or immature-form CD1d, respectively) or an isotype control monoclonal antibody (IgG2b). Immunoprecipitated proteins and whole cell lysates were western blotted with indicated antibodies. Equivalent amounts of whole cell lysate input in coimmunoprecipitations (Input) were loaded and blotted with antibodies against Strep tag, CD1d (CD1d.D5) or ER protein Grp94 for input controls. (C). Diagram of E protein domain structure and generation of E protein deletion mutants. (D). 293T.CD1d cells were transfected to express indicated E protein mutants and cell surface CD1d expression is examined by CD1d.51.1 anti-CD1d antibody staining followed by flow cytometry. (E). The relative levels of CD1d surface expression were plotted for indicated mutant E proteins. The relative CD1d expression level was calculated by dividing the MFI values of GFP-positive cells (transfected cells) by the MFI values of GFP-negative cells (untransfected cells). (F). 293T.CD1d cells were transfected to expressed indicated E protein deletion mutants and cell lysates were subjected to immunoprecipitation by anti-CD1d antibody, CD1d.51.1. Immunoprecipitated proteins were western blotted by anti-CD1d antibody, CD1d.D5 or anti-Strep antibody. Whole cell lysates (WCL) were also blotted by anti-CD1d antibody CD1d.D5, anti-Strep and anti-Grp94. (G). 293T.CD1d cells were transfected to express E protein CT construct and the transfected cells were subjected to MG132 or chloroquine treatment at indicated concentrations for 24 hours. Cells were harvested at 48 hours post transfection and lysates were blotted by anti-Strep antibody. (H). 293T.CD1d cells were transfected to express the TM domain of E protein. Transfected cells were lysed in 1% CHAPS and cell lysates were immunoprecipitated with anti-CD1d antibodies (CD1d.51.1 or CD1d.D5) or an isotype control (mIgG2b). Immunoprecipitants were blotted by anti-Strep antibody to detect co-immunoprecipitated TM domain and by anti-CD1d antibody CD1d.D5 to examine the CD1d protein levels. ****: p<0.0001 by unpaired Student’s t test.

To delineate the molecular and cellular mechanism of how E protein suppresses CD1d expression, we first examined potential interaction between E and CD1d proteins by co-immunoprecipitation. When cells were lysed using the detergent, Triton X-100, no interaction between the two proteins was detected (Fig 4A, first panel, last lane). However, using three different detergents, NP-40, Digitonin or CHAPS, to lyse the cells, co-immunoprecipitated E protein was clearly detected upon immunoprecipitation by anti-CD1d antibody CD1d.51.1 (Fig 4A, first panel). When comparing the immunoprecipitated CD1d proteins in the presence or absence of E protein expression, the level of CD1d protein was consistently lower in the presence of E protein expression (Fig 4A, second panel). CD1d protein is synthesized in the ER and exists in the cells in two forms [52–54]. About half of the newly synthesized CD1d molecules stay in the ER as an immature form, while the other half fold completely, associate with β2-microglobin, exit ER, acquire complex glycosylation and express on the cell surface as mature form [54,55]. The mouse monoclonal antibodies, CD1d.D5 and CD1d.51.1, recognize a linear epitope in the immature ER form and a conformational epitope in the mature and post-ER form of CD1d, respectively [52–54]. We repeated the co-immunoprecipitation experiment with these two monoclonal anti-CD1d antibodies using CHAPS to lyse cells (Fig 4B). Co-immunoprecipitation of CD1d with CD1d.51.1 monoclonal antibodies clearly demonstrated the interaction of E protein with the mature post-ER-form of CD1d. E protein also interacts with the ER-form of CD1d, but to a lesser extent, while an isotype control antibody minimally precipitated it (Fig 4B, first panel). Remarkably, the expression of E protein significantly led to decreased amount of mature CD1d (Fig 4A and 4B), but it does not reduce the level of the ER-form immunoprecipitated by CD1d.D5 antibody. This result suggested that the E protein functions to specifically degrade mature CD1d protein to reduce cell surface expression of CD1d. In whole cell lysate, the SDS-PAGE and western blot by CD1d.D5 antibody detected all CD1d proteins including the ER-form, which forms a distinct band due to their uniform molecule weight (fourth panel from top in Fig 4A and 4B), and the mature form, which appears as faint smear above the ER-form because of its complex glycans (fourth panel in Fig 4A and 4B). These mature forms of CD1d proteins can be readily detected when enriched in immunoprecipitated samples and showed a higher molecular weight due to its glycosylation (second panel in Fig 4B).

### 4. E protein downregulation of CD1d expression is mediated by its transmembrane domain

To dissect how E protein induces CD1d degradation and downregulates cell surface expression, we mapped domain(s) in E protein that are responsible for CD1d regulation. SARS-CoV-2 E protein is a 75-residue type II transmembrane protein composed of a short 8-a.a. N-terminus, 30-a.a. ion-conducting transmembrane domain and a 37-a.a. cytoplasmic tail [56]. We generated three constructs, consisting of transmembrane domain (TM) only, the transmembrane domain plus the N-terminal short peptide (N-TM), and the cytoplasmic tail (CT) (Fig 4C). Transient expression of individual constructs and flow cytometry showed that N-TM domain caused CD1d downregulation. However, TM domain downregulated CD1d equally (Fig 4D and 4E), suggesting that the N-terminal short peptide is not critical for this function. CT domain did not lead to any CD1d downregulation. However, it could not be detected by western blotting (Fig 4F). When we treated the transfected cells with the proteasomal inhibitor MG132, the CT domain became detectable (Fig 4G), suggesting that the CT domain was expressed but rapidly degraded by proteasomes. Chloroquine, which inhibits lysosomal proteolysis, had no effect. Previously it was shown that SARS-CoV E protein is ubiquitinated at the lysine 63 (K63) in its CT domain and leads to its degradation by proteasomal degradation [57]. This K63 residue is conserved in SARS-CoV-2 E protein, and it will be interesting to further examine whether this lysine is required for the ubiquitination and degradation of the CT domain.

Remarkably, similar to full-length E protein, the TM domain decreased the amount of mature CD1d, but has no effect on the ER-form immature CD1d (Fig 4H, second panel). The TM domain also interacted with mature CD1d protein based on co-immunoprecipitation, although it interacted with the immature form of CD1d comparably (Fig 4H, first panel). All these results suggested that TM domain was responsible for E protein-mediated CD1d degradation and downregulation.

### 5. SARS-CoV-2 E protein leads to degradation of mature CD1d protein in a proteasome and lysosome-dependent manner

**Fig 5:**
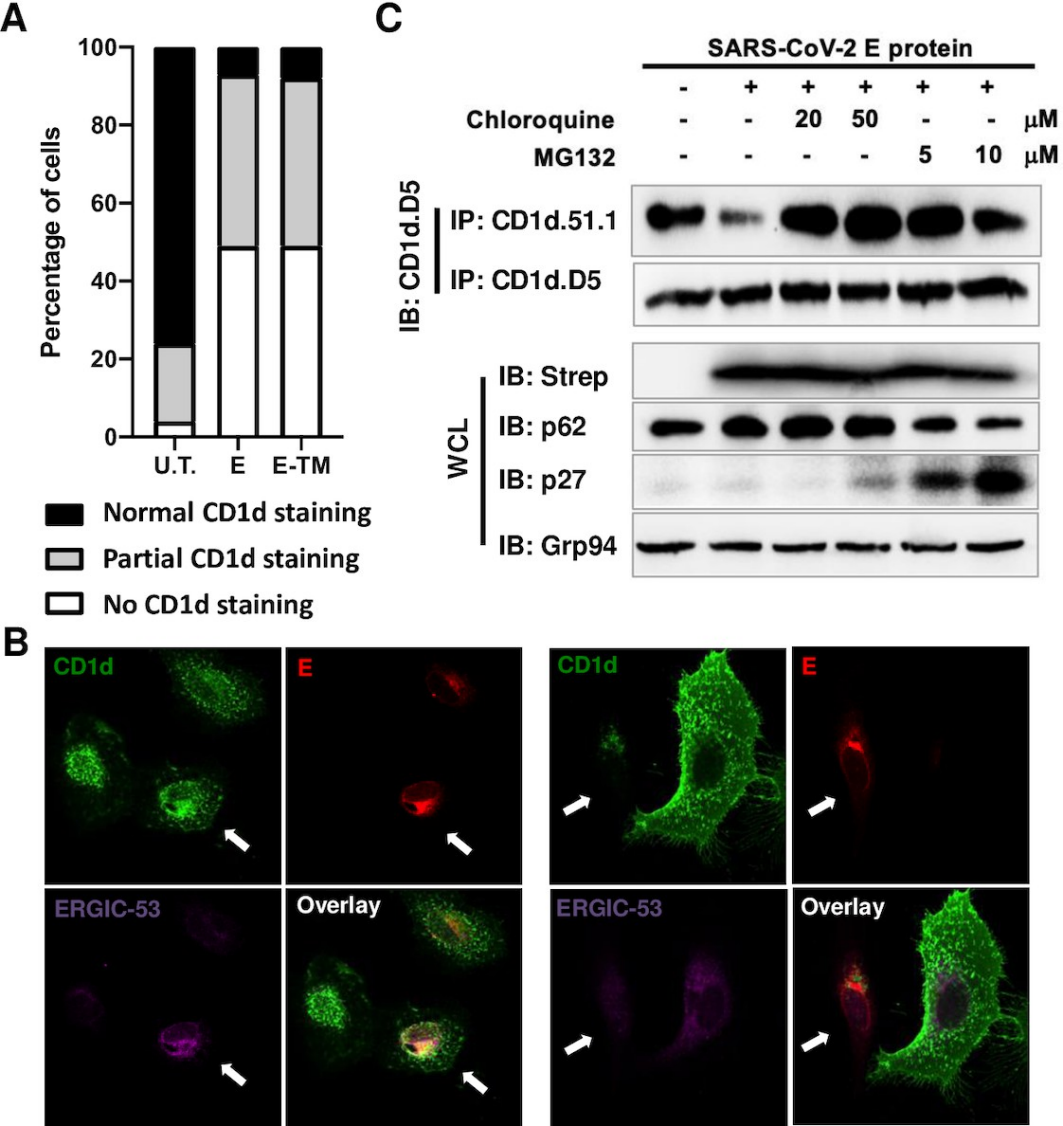
SARS-CoV-2 E protein leads to degradation of mature CD1d proteins in a proteasome- and lysosome-dependent manner. (A, B). HeLa.CD1d cells were transfected to express E protein. Forty eight hours post transfection, cells were fixed with 3.7% formaldehyde and subjected to co-staining with anti-CD1d (CD1d.51.1) and anti-Strep antibodies. Relative expression levels of mature CD1d protein is surveyed in HeLa.CD1d cells expressing either full-length E protein or its TM domain and compared to CD1d protein level in untransfected HeLa.CD1d cells (A). CD1d expression in HeLa.CD1d cells was categorized into three groups: normal CD1d staining, partial CD1d staining and no CD1d staining as defined in “Materials and Methods”. The percentages of HeLa.CD1d cells in each categorized group were presented. (B). Representative staining of mature CD1d and SARS-CoV-2 E protein in transfected cells expressing both E protein and CD1d. CD1d, Strep-tagged E protein and cellular ERGIC-53 proteins were co-stained and imaged. Arrows pointed to E protein-expressing transfected cells. (C). 293T.CD1d cells were transfected to express E protein and treated with MG132 or chloroquine at indicated concentrations. Cells were harvested 48 hours post transfection, lysed in 1% Triton and subjected to immunoprecipitation by anti-CD1d antibodies, CD1d.51.1 or CD1d.D5. Immunoprecipitants were blotted with anti-CD1d antibody (CD1d.D5). Whole cell lysates were blotted with p62 and p27 antibodies to verify the inhibition of lysosomal or proteasomal degradation. Grp94 protein was blotted as loading controls for all samples.

We then performed immunofluorescence staining of CD1d in E protein-expressing cells to examine the effect of E protein expression in individual cells, using HeLa.CD1d cells because of their superior imaging qualities. The cells were transfected with plasmids encoding SARS-CoV-2 E protein and co-stained for E protein and CD1d. In most transfected cells, mature CD1d protein was barely detectable by CD1d.51.1 antibody staining (Fig 5A), consistent with the results of western blotting. We tallied the transfected cells and categorized the remaining signal of CD1d staining into three groups, “normal CD1d staining” which showed CD1d staining signal as 70%-100% of average CD1d signal in untransfected cells, “partial CD1d staining” with 20%-70% of average CD1d signal, which clearly showed decreased CD1d staining or “no CD1d staining” with <20% of average CD1d signal, in which CD1d staining was barely detectable. In cells transfected with either full-length E protein or TM domain of E protein, the majority of cells (over 90%) showed clear reduction of CD1d staining. In some cells where the mature CD1d protein was detectable, we observed partial co-localization of CD1d with E protein in the ER-Golgi intermediate compartment (ERGIC) region (Fig 5B, left, arrow-pointed, [58]). However, in many other transfected cells, the mature CD1d protein was in much lower levels (Fig 5B, right, arrow-pointed), while in untransfected cells (E protein-negative), mature CD1d protein was localized mostly in the intracellular endosomal compartment and cell surface, as reported in previous studies [11,59] (Fig 5B, right, the untransfected cell was to the right of the transfected cell). Interestingly, in the transfected cells, the remaining CD1d protein was still co-localized with E protein in the ERGIC region (Fig 5B, right). These results suggest that the interaction with E protein relocated the mature CD1d protein to the ERGIC and eventually led to CD1d degradation in most transfected cells, which was consistent with the results from western blotting (Fig 4A and 4B).

To dissect whether E protein-mediated CD1d degradation is mediated by proteasomal or lysosomal pathways, we treated E protein-transfected 293T.CD1d cells with either MG132 or chloroquine. The p62 protein, also called sequestosome 1 (SQSTM1), is a ubiquitin-binding scaffold protein degraded by lysosomes. Its accumulation is commonly used as a marker for lysosome inhibition [60]. On the other hand, inhibition of proteasome-mediated degradation of proteins can interfere with the ordered and temporal degradation of cyclin-dependent kinase inhibitors (CKIs) such as p27Kip1 [61]. After treatment by MG132 or chloroquine, substantial accumulation of p27 and p62 proteins was confirmed by western blotting (Fig 5C), confirming the inhibition of proteasomal and lysosomal function, respectively. Under these conditions, either of the inhibitors could reconstitute the level of mature CD1d to that in untransfected cells (Fig 5C, first panel). As expected, neither of the treatments had impact on the level of immature CD1d proteins precipitated by antibody CD1d.D5 (Fig 5C, second panel). This result suggested that both proteasomal and lysosomal degradation were involved in the E protein-mediated degradation of mature CD1d proteins. Ubiquitination has been one of the major mechanisms for degradation of membrane proteins, either via polyubiquitination followed by proteasome-mediated degradation or monoubiquitination-mediated sorting of membrane proteins into lysosome-mediated degradation [62–64]. It will be of great interest to further investigate how these pathways are involved in E protein-mediated CD1d degradation and whether a ubiquitination-independent proteasomal degradation is involved, as suggested for degradation processes of other membrane proteins [65,66].

### 6. The putative ion channel function of E protein is required for its degradation of mature CD1d

**Fig 6:**
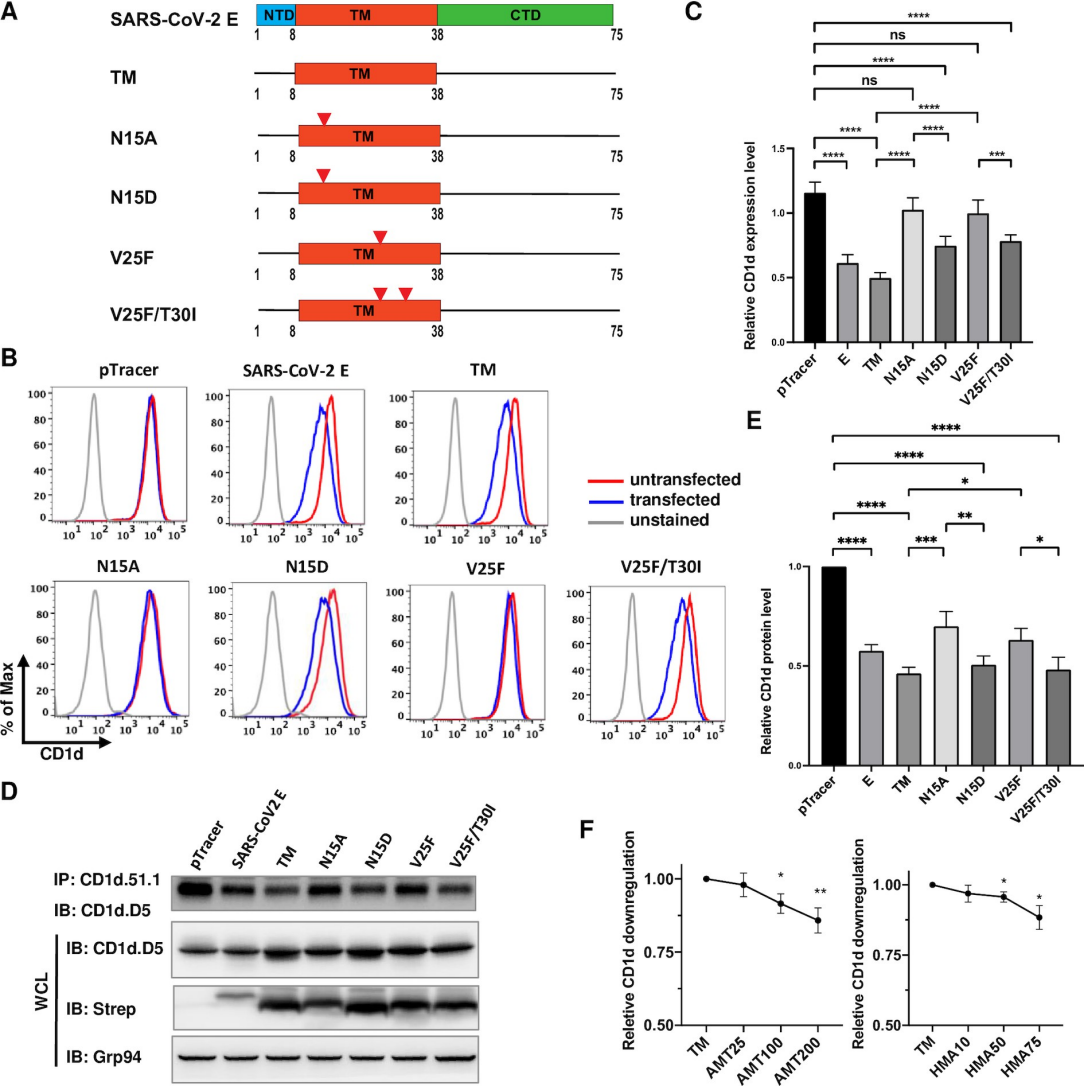
The putative ion channel function is required for E protein-mediated CD1d downregulation. (A). The domain structure of SARS-CoV-2 E protein and the mutagenesis schemes in its TM domain to ablate ion channel function (N15A and V25F) or to reconstitute ion channel function (N15D and V25F/T30I). (B, C). The effect of wild-type or mutant E proteins in downregulating cell surface CD1d levels in transfected 293T.CD1d cells was analyzed by flow cytometry. The relative CD1d expression level was calculated by dividing the MFI values of GFP-positive cells (transfected cells) by the MFI values of GFP-negative cells (untransfected cells). (D, E). 293T.CD1d were transfected with wild-type or indicated mutant E proteins and cell lysates were subjected to immunoprecipitation by anti-CD1d antibody (CD1d.51.1) and levels of mature CD1d protein were compared by western blotting. The relative CD1d protein level based on western blotting was calculated by dividing the chemiluminescence signal of CD1d protein in cells transfected with E gene or mutants by the chemiluminescence signal of CD1d protein in cells transfected with pTracer plasmid. (F). Ion channel inhibitors can revert E protein-mediated CD1d downregulation. 293T.CD1d cells were transfected to express the TM domain of E protein and treated with increasing concentrations of ion channel inhibitors, amantadine or hexamethylene amiloride (HMA). Forty-eight hours post transfection, cell surface CD1d expression was analyzed by flow cytometry. The relative CD1d downregulation upon treatment with ion channel inhibitors was calculated as described in “Materials and Methods”. Statistical analyses were performed by unpaired Student’s t test and one-way ANNOVA test (C-F). *, **, ***, ****: p<0.05, p<0.01, p<0.001, p<0.0001. ns: not significant.

Functionally, SARS-CoV-2 E protein belongs to a group of proteins called viroporins that are small and hydrophobic proteins and oligomerize in host cell membranes to form hydrophilic pores conducible for ions and small molecules [67]. The TM domains of coronavirus E proteins belong to the Class I subclass A viroporins [68]. Viroporins in this group contain M2 protein of influenza A virus and vpu protein of HIV [69,70]. These viroporins form homo-oligomers with the same topology, a short N-terminal ER luminal domain and a long cytoplasmic tail, whereas the subclass B viroporins, such as poliovirus P3A protein and human respiratory syncytial virus (RSV) SH protein, have the opposite topology [68]. An elegant study employing in vitro and in vivo viral evolution demonstrated that two essential residues, N15 and V25 in the TM domain of SARS-CoV E protein are essential for its viroporin function [71]. Furthermore, using serial passages in mouse infections, revertants and compensatory mutations were elegantly identified and characterized. Among them, revertant N15D from N15A mutant and compensatory T30I mutation for V25F mutant not only reconstituted the ion channel function of TM domain in vitro, but also restored the viral pathogenicity in vivo [71]. The TM domains of SARS-CoV and SARS-CoV-2 are completely conserved. We therefore investigated whether the putative ion channel function is critical for the CD1d down-regulation and degradation by mutating these two essential residues (Fig 6A). It was evident that mutating either of the two residues to alanine (N15A or V25A) completely abolished the CD1d downregulation (Fig 6A and 6B), while rescue/compensation mutants, including the N15D mutant or the V25A mutant with compensatory T30I mutation, reconstituted the CD1d downregulation (Fig 6B and 6C). Interestingly, total amounts of mature CD1d proteins, immunoprecipitated by CD1d.51.1 monoclonal antibodies, were correlated with the downregulation phenotype (Fig 6D and 6E). The mature CD1d levels were significantly lower in the cells that express wild-type or E proteins with revertant or compensatory mutations. On the other hand, there was no decrease in the amount of immature or ER-form CD1d protein in whole cell lysates (Panel 2 and Fig 6D). These results strongly suggested that the putative ion channel function was essential for CD1d degradation and decreased cell surface expression. To further test this hypothesis, we treated E protein-expressing cells with two different viroporin inhibitors, amantadine, a well-characterized viroporin inhibitor for influenza M2 viroporin and SARS-CoV-2 E protein [56,69] or hexamethylene amiloride (HMA), a specific inhibitor of another viroporin, vpu protein of HIV-1 [72]. 293T.CD1d cells were transfected with plasmids expressing E protein TM domain and immediately treated with either of the two inhibitors at various concentrations. Forty-eight hours post transfection, cells were stained and analyzed by flow cytometry to examine surface CD1d expression. Flow cytometry analysis of E protein-dependent CD1d downregulation clearly showed a dose-dependent relief of CD1d downregulation by both amantadine and HMA (Fig 6F), confirming the requirement of ion channel function for E protein-led CD1d downregulation.

### 7. Only E proteins of highly pathogenic coronaviruses possess the function of suppressing CD1d expression

**Fig 7:**
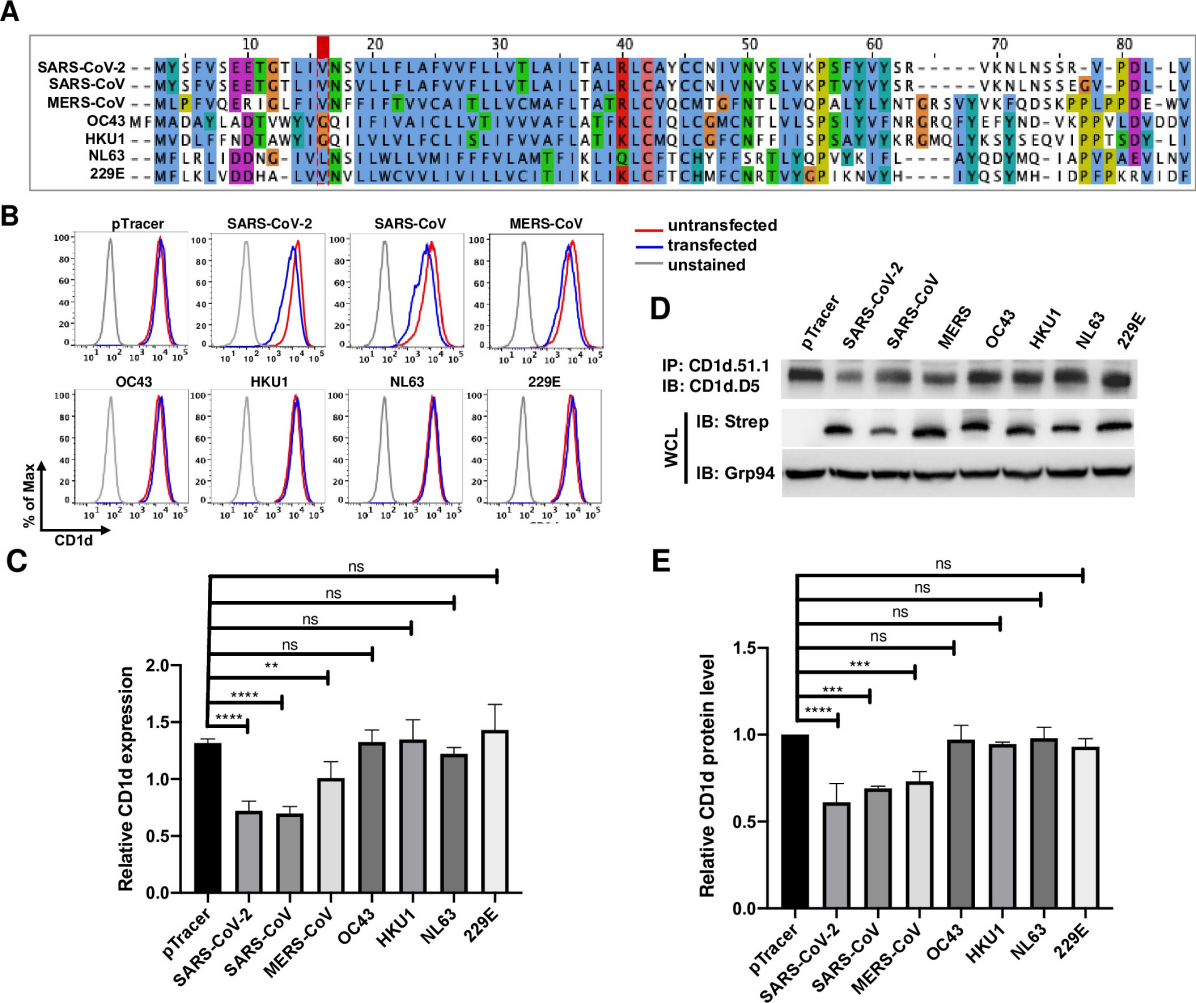
Only E proteins from highly pathogenic coronaviruses, but not common cold viruses downregulate CD1d expression. (A). Amino acid sequence alignment of E proteins from seven human coronaviruses. (B, C). 293T.CD1d cells were transfected to express individual E proteins and 48 hours later, subjected to flow cytometry to examine the CD1d surface expression level. (D, E). Lysates of transfected cells were subjected to immunoprecipitation by anti-CD1d antibody (CD1d.51.1) and levels of mature CD1d protein were compared by western blotting. Whole cell lysates were western blotted by anti-Strep antibodies to examine the expression of individual E proteins from human coronaviruses. The relative CD1d expression level and relative CD1d protein level were calculated as in Fig 6. Statistical analyses were performed by unpaired Student’s t test and one-way ANNOVA test (C-F). *, **, ***, ****: p<0.05, p<0.01, p<0.001, p<0.0001. ns: not significant.

To investigate how conserved the E protein evasion of CD1d function is among diverse human coronaviruses, we generated constructs expressing the E proteins from all seven human coronaviruses by optimizing the codon usage and chemically synthesizing the E genes (Fig 7). The most conserved region in E proteins is the TM domain, while the N-termini and CT domains have different degrees of conservation in different areas (Fig 7A). We transfected 293T.CD1d cells with plasmids expressing individual E proteins. Proper expression of these E proteins was verified by western blotting (Fig 7D, second panel). Remarkably, surface CD1d staining and flow cytometry demonstrated that only E proteins from the highly pathogenic coronaviruses, SARS-CoV-2, SARS-CoV and MERS, downregulated CD1d, while the E proteins from four human common cold coronaviruses, HCoV-OC43, HCoV-229E, HCoV-NL63 and HCoV-HKU1, had no effect on the levels of CD1d surface expression (Fig 7B and 7C). Consistently, decrease of mature CD1d protein levels was only detected in cells expressing E proteins from highly pathogenic coronaviruses, but not common cold coronaviruses (Fig 7D, top panel, and Fig 7E). This apparent correlation of inhibiting CD1d expression with the severity of coronaviruses is consistent with our hypothesis that E protein evasion of CD1d and NKT cell function is an important virulence factor in coronavirus pathogenesis.

### 8. NKT cell activation overcomes SARS-CoV-2 evasion of NKT cell function and mitigates disease severity

**Fig 8:**
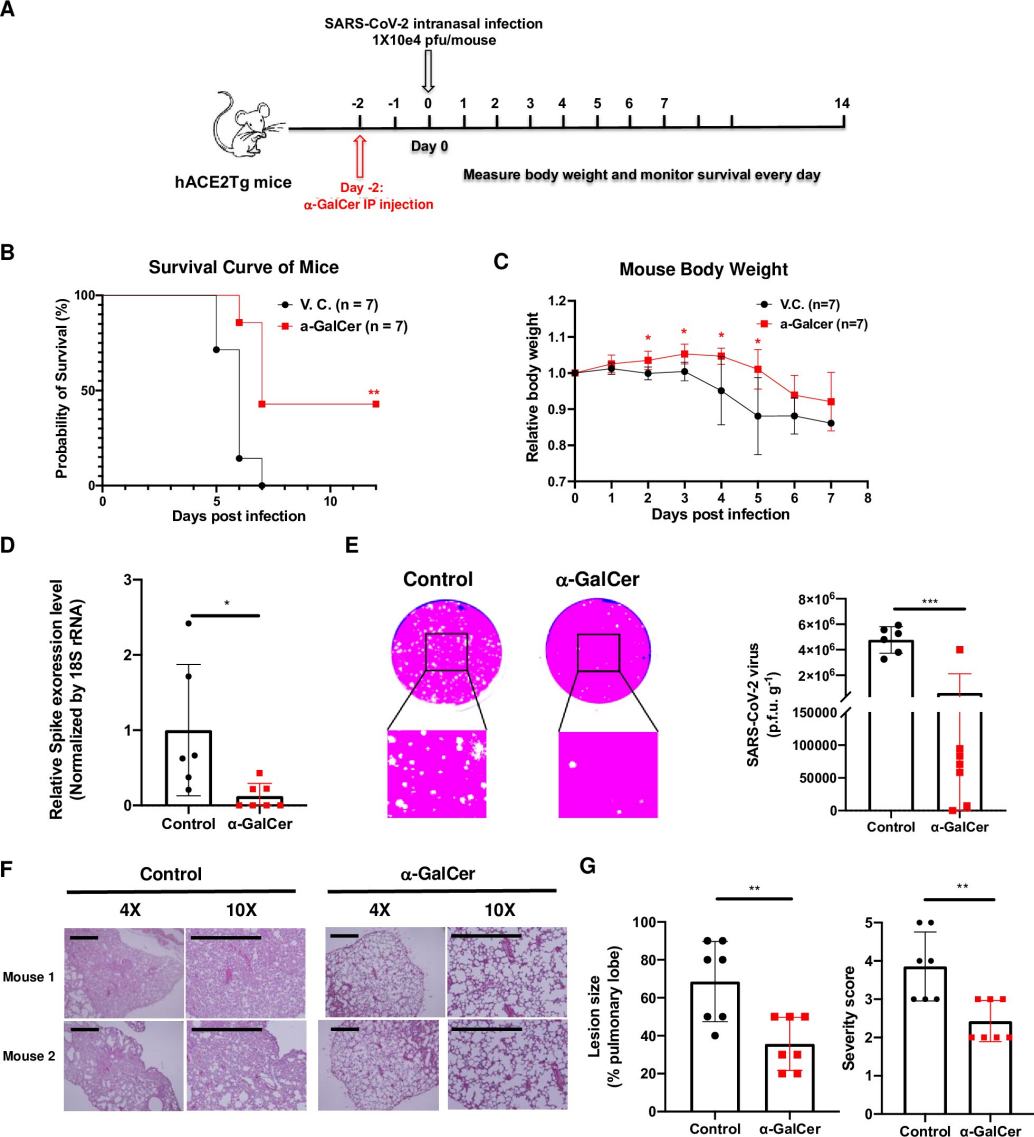
iNKT cell activation overcomes SARS-CoV-2 immune suppression of NKT cell function, mitigates disease severity and improves host survival in mice. (A). A schematic presentation of SARS-CoV-2 infection in mice. (B, C). K18-hACE2-Tg mice (n = 7 per group) were administered once with α-galactosylceramide (α-GalCer, 2 μg per mouse) and two days post treatment, were intranasally infected with SARS-CoV-2 (Isolate USA-WA1/2020, 1X10e4 pfu per mouse). (D-G). iNKT cell activation reduced viral replication and disease severity in lung tissues. (D). Local virus replication analyzed by real-time PCR for mRNAs of viral spike proteins using lung tissue RNA samples on day 3 (n = 6 for control group and n = 7 for α-GalCer-treated group) from infected K18-hACE2-Tg mice untreated (control) or pre-treated with α-GalCer 2 days prior (α-GalCer). (E). Titration of local viral load by plaque assays using the supernatant of homogenized lung tissues on day 3 (n = 6 for control group and n = 7 for α-GalCer-treated group). (F). Histopathology analysis of formalin-fixed and hematoxylin and eosin (H&E)-stained lung tissues (n = 7 per group). (G). Comparison of pulmonary lesion sizes and pathological severity scores in vehicle control and α-GalCer-treated mouse groups (n = 7 per group), based on the percentage of affected area in lung tissues (see “Materials and Methods”). Representative images of plaque assay (E) and H&E staining (F) were presented. Scale bar, 250 μm (F). B-G were representative data from three independent experiments and relative body weight (C) was plotted as the mean ± standard deviation (s.d.). D-G were representative data of three independent experiments and D, E and G were plotted as the mean ± s.d. Statistical analyses were performed using Mantel-Cox log-rank test (B), two-way ANOVA followed by Sidak’s post-test (C) and two-side unpaired Student’s t-test (D, E, G). * p<0.05; ** p<0.01; ***p<0.001.

To antagonize SARS-CoV-2 immune evasion of NKT cell function, we explored the potent glycolipid ligand of, α-galactosylceramide (α-GalCer) to activate iNKT cells and examined the potential antiviral effect of these glycolipids during SARS-CoV-2 infection. Two micrograms of α-GalCer were administered to K18-hACE2-Tg mice (n = 7, male and female) and two days later the mice were infected with SARS-CoV-2 (Isolate USA-WA1/2020, 1X10e4 pfu per mouse) (Fig 8A). In control mice infected with the virus, the infection led to up to 15% decrease in body weight within 6 days post infection and all mice died by day 7 post infection (Fig 8B and 8C). On the other hand, in treated mice, on average there was significantly less loss in body weight. Approximately 40% of treated mice survived the virus challenge (Fig 8B). These results strongly suggested that activated iNKT cells could exert potent antiviral function and the viral evasion of iNKT cell function could be overcome by potent iNKT cell ligands. To investigate the antiviral mechanism by the iNKT cell ligand, we measured viral replication in infected mice by real-time PCR analysis of viral gene expression as well as viral loads, assessed by plaque assays, in the lungs of infected mice. Both expression levels of the spike protein gene and viral titers in the lung were significantly lowered in the α-GalCer-treated mice (Fig 8D and 8E), suggesting a lower level of viral replication. Furthermore, histology examination of mouse lung tissues demonstrated reduced inflammation and smaller lesions of viral infections (Fig 8F and 8G). Consistent with these results, the disease scores were significantly lower in α-GalCer-treated mice (Fig 8G).

**Fig 9:**
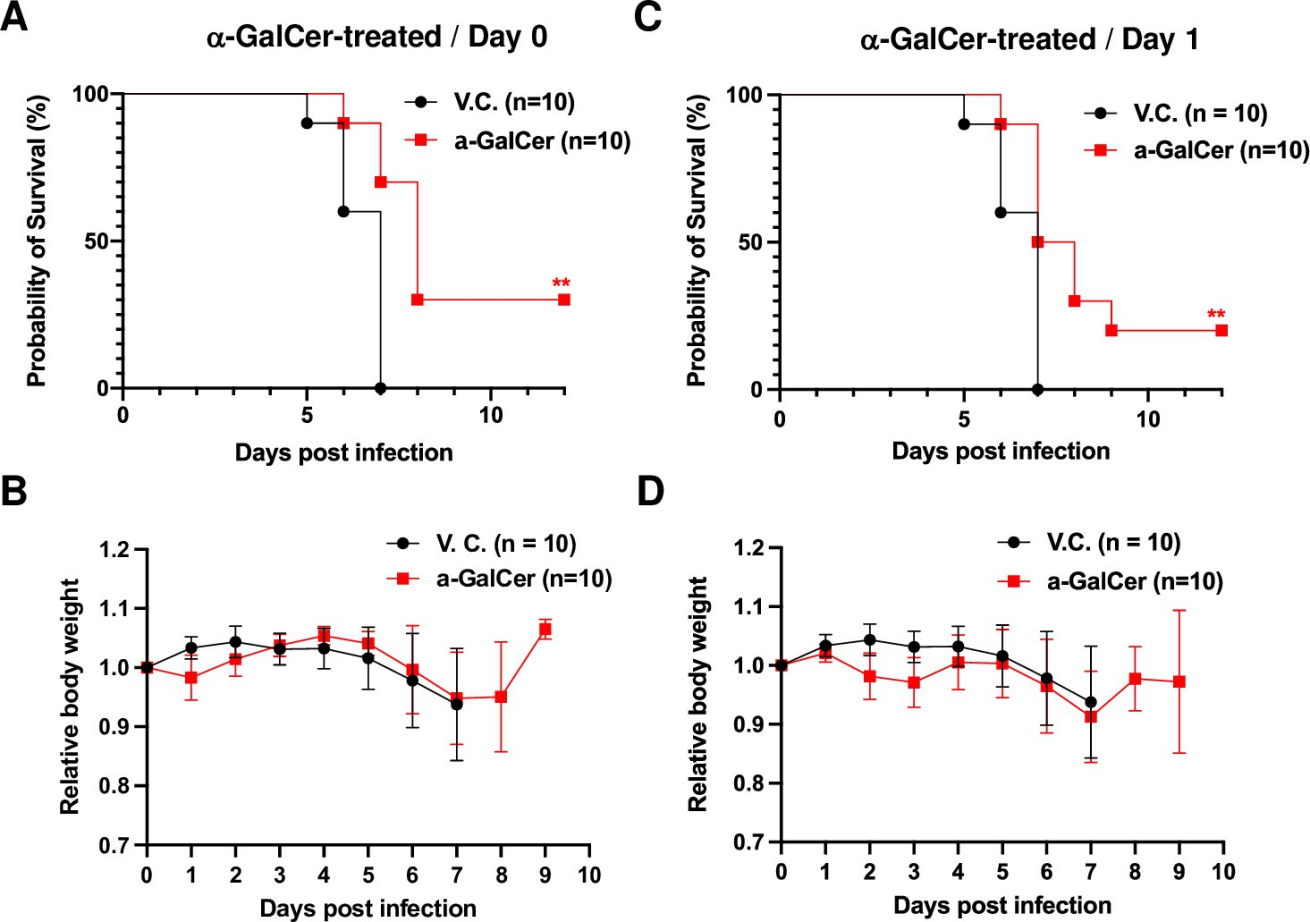
Therapeutic activation of iNKT cells post SARS-CoV-2 infection can mitigate disease severity and improve host survival in mice. K18-hACE2-Tg mice (n = 10 per group) were infected with SARS-CoV-2 (Isolate USA-WA1/2020, 1X10e4 pfu per mouse) then immediately administered with α-GalCer (2 μg per mouse) (A, B) or administered once with α-GalCer (2 μg per mouse) at one day post infection (C, D). Representative data of three independent experiments were presented. Infected mice were monitored daily for body weight and disease severity. Differences in animal survival were evaluated with Mantel–Cox log-rank test. ** p< 0.01.

The administration of α-GalCer prior to viral infection represented a prophylactic procedure. To investigate whether iNKT cell ligands have a therapeutic effect, α-GalCer was immediately administered after the initiation of viral infection. Mouse survival was again significantly improved for all treated mice (Fig 9A), although the overall survival rate was lower than that in mouse group pre-treated with α-GalCer (Fig 8B). Loss of body weight was also mitigated in treated group to some degree (Fig 9B). To further investigate whether there is a therapeutic window after initiation of SARS-CoV-2 infection, we repeated the infection experiment, in which α-GalCer was injected one day post viral infection. A significantly improved survival was still detected in most of the mice (Fig 9C), even though not as much as that in mice treated with α-GalCer on Day 0 and the body weight loss was not improved significantly (Fig 9D). All these results demonstrated that iNKT cell activation could mitigate viral pathogenesis during severe SARS-CoV-2 infection and improve the host survival.

**Fig 10:**
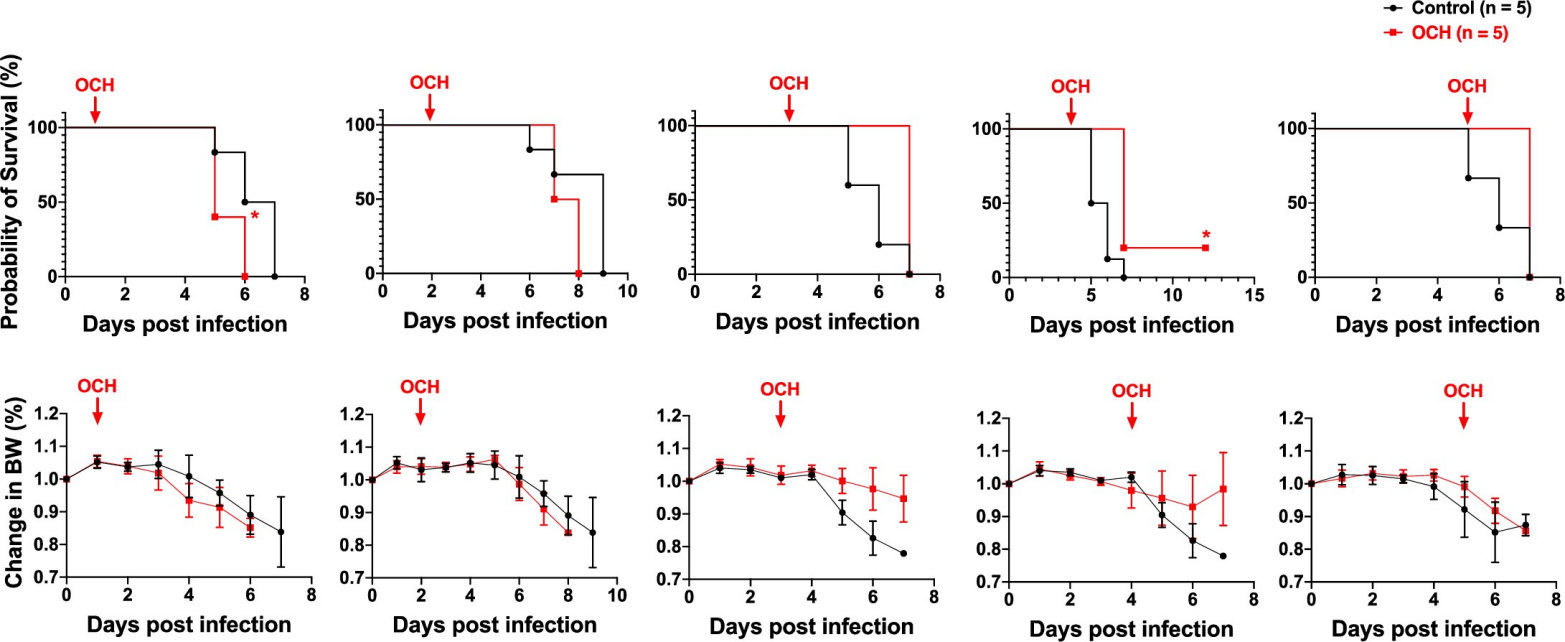
α-GalCer analog, OCH, only demonstrates therapeutic benefits in late stages of SARS-CoV-2 infection. hACE2-Tg mice (n = 5) were infected with SARS-CoV-2 (Isolate USA-WA1/2020, 1X10e4 pfu per mouse). Three micrograms of OCH glycolipids were administered to infected mice at different time points post infection, from day 1 to day 5. Mouse body weight (BW) changes and survival were tallied and compared. Statistical analyses of mouse survival were performed using Mantel-Cox log-rank test. * p<0.05.

For future clinical application of iNKT cell ligands, it will be informative to define their therapeutic window. hACE2-Tg mice represent a mouse model for severe SARS-CoV-2 infection under the current condition of experimental infection [73]. Therefore the disease course is rather short with all mice dying within 7 days (Fig 8). When we treated the infected mice further in the infection course at two days post infection, we detected minimal therapeutic effect. We reason that treating with the mostly Th1-biased α-GalCer towards late stage of infection may actually deteriorate the disease severity due to the hyperinflammation in late infection [74]. To test our hypothesis, we utilized a Th2-biased α-GalCer analog, OCH [75,76]. The OCH glycolipid was administered to infected mice at different time points, from day 1 to day 5. Remarkably, early administration of OCH in the first two days post SARS-CoV-2 infection, actually deteriorated the viral diseases and led to earlier mouse death (Fig 10), presumably due to the dampening of antiviral immune responses. This regulatory iNKT cell ligand only demonstrated therapeutic benefits from day 3, with day 4 showed the highest therapeutic benefit (Fig 10). One out of five mice survived the deadly infection. It will be very interesting to investigate whether OCH is improving the disease outcome via mitigating the hyperinflammation in late stages of SARS-CoV-2 infection [74].

### 9. NKT cells are essential role for optimal antiviral immune responses in SARS-CoV-2 natural infection

**Fig 11:**
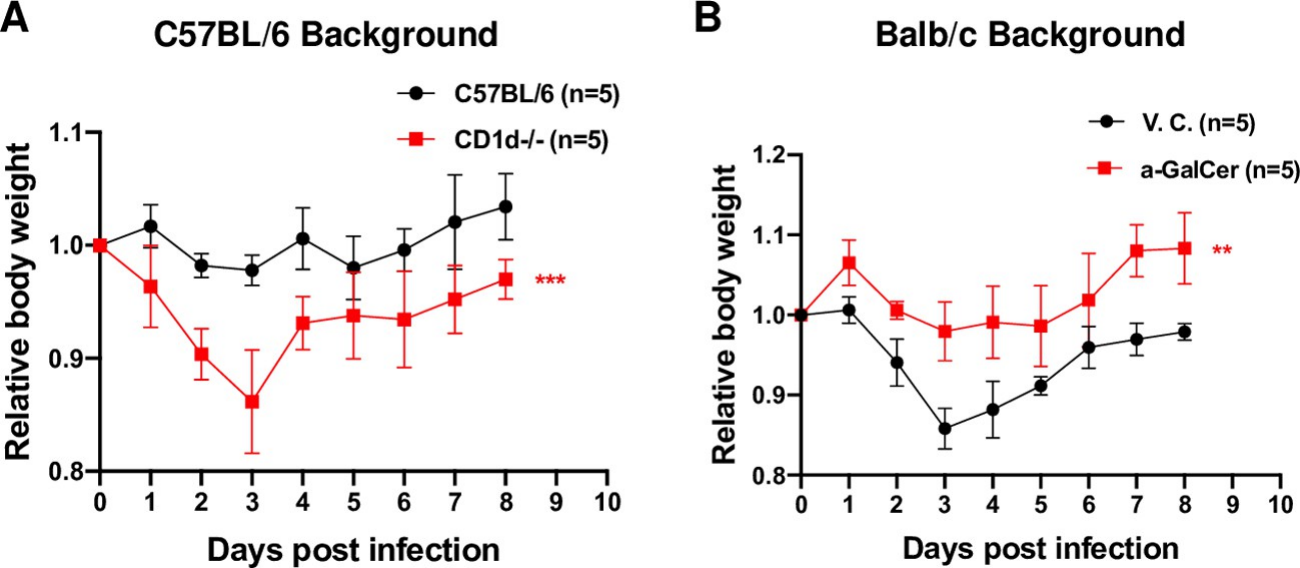
NKT cells are essential for optimal anti-SARS-CoV-2 immune responses and activation of iNKT cells can mitigate diseases caused by SARS-CoV-2 infection. (A). C57BL/6 or mCD1d-knockout (CD1d-/-) mice (n = 5 per group) were infected with mouse-adapted virus CMAp20 strain (1X10e6 pfu/mouse). (B). Balb/c mice (n = 5 per group) were infected with CMAp20 strain (1X10e6 pfu/mouse) then treated with vehicle control (v.c.) or α-GalCer (2 μg/mouse). Mouse body weight was monitored daily. Statistical analyses were performed using two-way ANOVA followed by Sidak’s post-test. ** p<0.01; ***p<0.001.

While we have demonstrated that pharmacologically activating iNKT cells could mitigate the disease severity by SARS-CoV-2 infection in hACE2-Tg mice, it is currently unknown whether iNKT or all NKT cells are essential for anti-SARS-CoV-2 immune responses during natural infection. To address this question, we employed mouse-adapted viruses recently developed. In the first two years of Covid-19 research, most in vivo SARS-CoV-2 research used the hACE2-Tg mice, which provided large amount of valuable information regarding the SARS-CoV-2 pathogenesis. This was the mouse line we have used for most of our studies. Nevertheless, mouse-adapted viruses have been recently developed to overcome some limitations of hACE2-Tg mice and better recapitulate the SARS-CoV-2 pathogenesis in human [77–79]. Therefore, we employed the mouse-adapted viruses to address the requirement of NKT cells for anti-SARS-CoV-2 immunity. Mouse-adapted (MA) viruses (CMAp20 strain) were used to infect wild-type C57BL/6 or CD1d-knockout mice (1X10e6 pfu/mouse). The CD1d-knockout mice lack all NKT cells [80]. Remarkably, we detected significantly more loss of body weight in mice lacking NKT cells (Fig 11A), suggesting that NKT cells are essential for optimal anti-SARS-CoV-2 immunity. While MA viruses caused minimal disease or loss of body weight in C57BL/6 mice, the infection caused up to 15% body weight loss in CD1d-knockout mice, peaked at Day 3. Consistent with the report with another mouse-adapted virus strain generated independently [79], the CMAp20 mouse-adapted virus demonstrated stronger pathogenicity in Balb/c mice than that in C57BL/6 mice (Fig 11A and 11B). Also consistent with the published report [78], in healthy young Balb/c mice (< 6 months old), infection by up to 1X10e6 pfu per mouse did not lead to mouse death, except severe body weight loss (15–20% body weight). To examine whether therapeutic activation of iNKT cells can mitigate the SARS-CoV-2 pathogenesis, we performed iNKT cell activation in Balb/c mice as the potential therapeutic benefit might not be manifested in resistant wild-type C57BL/6 mice. α-GalCer-treated Balb/c mice barely lost any body weight compared to that in vehicle control-treated mice (Fig 11B). All these results supported the essentialness of NKT cells in anti-SARS-CoV-2 immunity as well as the effectiveness of iNKT cell ligands as therapeutic agents.

## Discussions

### 1. Evasion of CD1d antigen-presentation and NKT cell function by SARS-CoV-2

Viral suppression of NKT cells via downregulation of CD1d antigen presentation has been well documented for both DNA and RNA viruses including HIV, HSV-1, KHSV and other viruses [8,11,13]. It has also been extensively studied how SARS-CoV and SARS-CoV-2 suppress the innate immunity, in particular, the production and signaling pathways of type I interferon pathways [81]. Several nonstructural and structural proteins target diverse cellular factors in these pathways. Therefore, it may not be surprising that highly pathogenic SARS-CoV and SARS-CoV-2 viruses have evolved to inhibit CD1d expression and innate-like NKT cell function. It is currently unknown how this new immune evasion mechanism leads to blocking or suppressing of other immune cell function downstream of NKT cells partially due to the limited information on how NKT cells function in antiviral immunity. Nevertheless, the direct inference from our study is that SARS-CoV-2 can infect professional antigen-presenting cells such as DCs and at least partially inhibit the activation of NKT cells in peripheral lung tissue, during DC trafficking to or within the lymph nodes. This immune evasion may weaken the immune-stimulating function of NKT cells early in the infection and allow more efficient viral replication and/or pathogenesis. Importantly, CD1d is expressed not only in professional antigen-presenting cells, but also in airway and respiratory epithelial cells [82]. The CD1d expression in these epithelial cells has been suggested to be involved in rapid activation and expansion of local NKT cells [83]. Many of these epithelial cells express the major SARS-CoV-2 receptor, ACE2 and are susceptible for viral infection. Therefore, we reason that SARS-CoV-2 infection of these epithelial cells and suppression of CD1d expression may lead to reduced local NKT cell activation and benefit viral replication.

We identified E protein of SARS-CoV-2 specifically downregulates CD1d expression in antigen-presenting cells and inhibits the activation of NKT cells. To further dissect the exact mechanism how this putative immune evasion mechanism of SARS-CoV-2 enhances viral pathogenesis, a mutant virus lacking E protein will be instrumental to compare the functional status of NKT cells and its effector function on other immune components. E protein is an important viral structure protein and is required for optimal viral pathogenesis in vivo [84], therefore an E protein-deficient virus is expected to present reduced fitness and/or virulence. A mutant with specific ablation of CD1d downregulation function will be desirable. Such a mutant (E:N15A) has been reported for the highly homologous SARS-CoV virus [71]. It is remarkable that, while the viral replication is not impeded significantly in vitro or in vivo, the mutant virus is significantly attenuated in virulence in vivo [71]. The rescue virus (E:N15D), on the other hand, is similarly virulent as the wild-type virus. It is tempting to hypothesize that the absence of CD1d downregulation and NKT cell evasion during infection by E:N15A mutant virus is at least partially responsible for its reduced virulence in vivo.

### 2. The role of putative ion channel function in SARS-CoV-2 E protein-mediated CD1d downregulation

Our results suggested that the putative ion channel function of SARS-CoV-2 E protein is required for its downregulation of CD1d expression. More work needs to be done to delineate the exact molecular mechanism of how the ion channel function of E protein leads to CD1d degradation, possibly via lysosome, proteasome or both lysosome and proteasome-mediated degradation (Fig 5C). One report investigating the molecular mechanism for SARS-CoV E protein downregulation of another cell surface protein, γ-epithelial sodium channel (γ-ENaC) demonstrated that the decrease of total protein levels and surface expression of γ-ENaC requires specific activation of PKCζ(PKC-zeta) [85]. It is interesting, in this regard, that multiple reports have supported a role of PKCζ [86] and other PKC members (PKCδ and PKCθ) in regulating CD1d expression [87–89]. It is tempting to hypothesize that SARS-CoV-2 E protein activates PKCζ and degrades CD1d to downregulate its surface expression. It will be interesting to investigate whether and how the ion channel function of SARS-CoV-2 E protein can lead to PKCζ activation. Nevertheless, if the PKCζ activation is the key mechanism for CD1d degradation and downregulation, it will be important to compare the capacity of E proteins from different coronaviruses in activating PKCζ and delineate whether this capacity is correlated to their function in downregulation of CD1d expression.

### 3. The antiviral roles of iNKT cells during anti-SARS-CoV-2 immune responses

Activation of iNKT cells by α-GalCer significantly mitigated the severity of SARS-CoV-2 infection (Fig 8). Currently, it is unknown whether this antiviral effect is mediated by direct cytolytic effect of activated iNKT cells as reported in EBV-infected lymphoblastoid cell lines [90] and other bacterial pathogens [91,92], or via indirect activation of other immune cells including NK, macrophage and CD8+ T cells for their cytolytic function [5,29]. Nevertheless, since the activated iNKT cells suppress viral replication and pathogenesis through a mechanism different from the most widely used antivirals, including the major protease inhibitor, nirmatrelvir, a future combination therapy will have potentials for a more efficacious treatment.

Our therapeutic exploration of both Th1-biased α-GalCer and its Th2-biased analogs suggested that Th1-type cytokines secreted by activated iNKT cells early in the infection can enhance antiviral immunity. It will be therefore interesting to explore more potent Th1-biased iNKT cell ligands, such as 7DW8-5 [93] and Nu-α-GalCer [94] for early treatment of SARS-CoV-2 infections. On the other hand, more Th2-biased α-GalCer analogs, such as C20:2 α-GalCer [95], may be harnessed to mitigate the hyperinflammation in severe Covid infections to improve disease outcome.

## Materials and methods

### Ethics statement

All animal procedures were performed at ABSL3 facility of Keck School of Medicine at University of Southern California and approved by the Institutional Animal Care and Use Committee (IACUC) and the Institutional Biosafety Committee (IBC) of the University of Southern California.

### Viruses, cells, antibodies and plasmids

The SARS-CoV-2 (Isolate USA-WA1/2020) was obtained from BEI, propagated and titered in Vero-E6 cells as previously described [96]. The mouse-adapted virus CMAp20 strain was generously provided by Dr. Pei-Yong Shi at University of Texas Medical Branch at Galveston. The Vα14/Vβ8.2 iNKT cell hybridoma cell lines DN32.D3 and Hyb1.2 were kindly provided by Dr. Albert Bendelac (University of Chicago, Illinois) and Dr. Mitch Kronenberg (La Jolla Institute for Allergy and Immunology, La Jolla, California), respectively. The iNKT cell hybridoma cell lines, KI-2, KI-15, KI-16 and KI-17 were generated from human CD1d-knock in mouse [97] and generously provided by Dr. Steven Porcelli (Albert Einstein Medical College, New York, NY). The 293T. CD1d and HeLa.CD1d cell lines were generated by retroviral transduction and have been described previously [54,98]. HeLa.CD1d.hACE2 cells were generated by transducing HeLa.CD1d cells with retroviral expressing human ACE2 (hACE2) gene and provided by Dr. Joanne Pawlak at Yale University School of Medicine. Bulk HeLa.CD1d.hACE2 were sorted for hACE2 expression and used for SARS-CoV-2 infection. In vitro derivation of human primary dendritic cells from human peripheral blood mononuclear cells as described previously [11]. Human peripheral blood from an anonymous healthy donor was acquired from Children’s Hospital Los Angeles Blood Donation Center following a protocol approved by University of Southern California Institutional Review Board (IRB) and Institutional Biosafety Committee (IBC). Monoclonal anti-CD1d antibodies, CD1d.D5 and CD1d.51.1, were generously provided by Dr. Steven Balk at Harvard Medical School and Dr. Steven Porcelli at Albert Einstein Medical College, respectively. Rabbit antibodies against ERGIC-53 (Abcam), p62 (ProteinTech), p27 (Cell Signaling) and SARS-CoV-2 nucleocapsid (N) protein (FabGenni), mouse antibodies against Strep tag (BioLegend), CD1a, CD1b, CD11b, CD11c, CD14, CD45 (clone 104), HLA-A,B,C (W6/32), CD71, CD63, Lamp1/CD107a and HLA-DR from BD, and rat antibodies against Grp94 (Enzo Life Sciences), CD1d (1B1, BD) and CD16/32 (clone 93, ThermoFisher) were used following manufacturer’s instructions. The SARS-CoV-2 expression library was generously provided by Dr. Nevan Krogan (University of California San Francisco) [51].

Deletion mutants of SARS-CoV-2 E protein were generated based on solved 3-D structure of its TM domain [56]. The N-terminal amino acids 1–38 (N-TM) or 9–38 (TM), or C-terminal 39–75 (CT) were PCR amplified, fused with two Strep tags and cloned into pLVX vector as in the original E protein construct [51]. To generate E protein mutants lacking ion channel function [71], N15 or V25 was mutated to alanine using PCR-based mutagenesis. To generate rescue mutants, N15 was mutated to aspartic acid while compensatory mutation T30I was generated on the background of V25F mutant to reconstitute the ion channel function of E protein [71]. All constructs were sequenced to verify the desired mutations.

For expression of E proteins from all seven human coronaviruses, E protein sequences from SARS-CoV (UniProt #P59637), MERS (UniProt # K9N5R3), HCoV-OC43 (UniPort # Q04854), HCoV-HKU1 (UniProt# Q5MQC8), HCoV-NL63 (UniProt# Q6Q1S0), HCoV-229E (UniProt# P19741) were converted to DNA sequence with optimized codon usage in human cells, fused to 2X Strep tags as in SARS-CoV-2 E protein construct [51], chemically synthesized (Genewiz) and cloned into pLVX vector (Clontech). The entire DNA constructs were Sanger sequenced to verify correct amino acid sequences. pLPCX.mCD1d plasmid expressing mouse CD1d was generated by subcloning mouse CD1d into BamHI/EcoRI site of pLPCX from pBabe.mCD1d plasmid [99] generously provided by Dr. Sebastian Joyce of Vanderbilt University.

### Screening of SARS-CoV-2 expressing library for viral gene(s) downregulating CD1d expression

293T.CD1d cells were co-transfected with individual pLVX constructs expressing SARS-CoV-2 genes [51] and pTracer-EF/V5-His A (Invitrogen) by polyethylenimine (PEI) as previously described [59]. Forty-eight hours post transfection, cells were harvested and stained with anti-CD1d antibody CD1d.51.1 followed by PE-conjugated goat anti mouse secondary antibody (BioLegend) and analyzed by flow cytometry. GFP-positive and GFP-negative cells were designated as transfected and untransfected cells, respectively.

### Effect of ion channel inhibitors on E protein-mediated CD1d downregulation

293T.CD1d cells were co-transfected with the pLVX construct expressing SARS-CoV-2 E protein and pTracer-EF/V5-His A by polyethylenimine (PEI) and immediately treated with either vehicle control (solvents used, water for amantadine and DMSO for hexamethylene amiloride) or indicated concentrations of inhibitors. Forty-eight hours post transfection, cells were harvested and stained with anti-CD1d antibody CD1d51.1 followed by flow cytometry. To calculate the relative level of CD1d downregulation, the rate of CD1d downregulation at each treatment concentration was calculated by dividing the reduced CD1d mean fluorescence intensity (MFI) (CD1d MFI in untransfected cells minus CD1d MFI in transfected cells) by CD1d MFI in untransfected cells. Then the relative level of CD1d downregulation was calculated as dividing the rates of CD1d downregulation at indicated inhibitor concentrations by the rate of CD1d downregulation in vehicle control-treated cells.

### Mouse strains and in vivo SARS-CoV-2 infection

C57BL/6 and K18-hACE2-Tg (B6.Cg-Tg(K18-ACE2)2Prlmn/J) mice were purchased from the Jackson Laboratory (Bar Harbor, ME) and bred locally. K18-hACE2 transgene was maintained at hemizygous status in experimental mice. CD1d-knockout mice in C57BL/6 background were generously provided by Dr. Chyung-Ru Wang at Northwestern University in Chicago, USA. For SARS-CoV-2 infection, 1X10e4 pfu/mouse wild-type (Isolate USA-WA1/2020) or 1X10e6 pfu/mouse mouse-adapted (CMAp20 strain) viruses were inoculated intranasally per mouse. Mice were monitored every day for weight loss and survival. Liver mononuclear cells (LMNCs) were prepared using Percoll gradients as previous described [97]. For single cell preparation of lung tissues, mouse lungs were minced, ground and passed through 70 μM filters. Red blood cells were lysed in ammonium chloride-containing buffer as previously described [97] and then single cell suspension was subjected to staining with α-GalCer-CD1d tetramer (NIH tetramer facility) staining. For iNKT cell activation, two micrograms of α-GalCer (Funakoshi Inc.) or three micrograms of OCH (NIH tetramer facility) were administrated to mouse intraperitoneally and the mice were infected after two days.

### Quantitation of SARS-CoV-2 viral replication in mice and pathological analysis

To quantitate viral replication, real-time PCR analysis of viral spike protein gene was performed as previously described [96]. Total RNA was collected from infected cells or mouse tissues at indicated time point using TRIzol reagent (Thermo Fisher Scientific) according to manufacturer’s instructions. Then, 1 μg of total RNA was used for RT–PCR using the SuperScript IV Reverse Transcriptase (Thermo Fisher Scientific) and random hexamers. Complementary DNA was diluted by 1:10 and 1 μl diluted cDNA was used for qPCR analysis. The qPCR analysis was performed on the Bio-Rad CFX Connect 96-well Real-Time qPCR module system with iTaq Universal SYBR Green Supermix (Bio-Rad). Each sample was run in three or four technical replicated wells. One primer set (SC2-S-F: GCTGGTGCTGCAGCTTATTA; SC2-S-R: AGGGTCAAGTGCACAGTCTA) targeting SARS-CoV-2 virus spike protein was used to measure the relative SARS-CoV-2 viral RNA and normalized to a mouse 18S rRNA primer set (18S rRNA-F, GTAACCCGTTGAACCCCATT; 18S rRNA-R: CCATCCAATCGGTAGTAGCG) for mouse samples. A spike protein expression plasmid was used to generate the standard curve of absolute quantification PCR analysis. The results were analyzed using software QuantStudio Software v.1.3 (Thermo Fisher Scientific). Each assay was performed in triplicate with four technical replicates and each assay included no-template negative controls.

For histological analysis, fresh lung tissues were fixed with 10% formalin for 24 hours and then processed in University of Southern California School of Pharmacy Histology Laboratory H&E staining. Slides were evaluated by a pathologist under a microscope and fields were randomly selected for analysis. Pictures were taken at ×4 and ×10 magnification using the Olympus BX61 microscope along with DP71 digital camera (Olympus). For histopathology score in infected mice, after H&E staining, the size of pulmonary lesions was determined by the mean percentage of affected area in each section of lobes from each animal. Lung tissues were then scored on a 0–4 system for pathological severity in these infected mice: 0, no pathological change; 1, affected area (≤10%); 2, affected area (≤30% and >10%); 3, affected area (≤60% and >30%); and 4, affected area (>60%).

## Statistical analysis

Descriptive statistics, including means and standard deviation (s.d.), were computed for each group. Data were presented as mean ± s.d. unless otherwise indicated. The sample size for each experiment is included in the figure legends. Statistical analyses were performed with unpaired Student’s t-test, one-way ANOVA test and two-way ANOVA followed by Sidak’s post-test, as indicated in figure legends. Differences in animal survival were evaluated with Mantel–Cox log-rank test. *P < 0.05, **P < 0.01, ***P < 0.001 and ****P < 0.0001. NS denotes not significant (P value >0.05). All analyses were performed with GraphPad Prism (GraphPad Software).

**Transient transfection, cell lysis, immunoprecipitation, co-immunoprecipitation, SDS-PAGE, western blotting, immunofluorescence, flow cytometry, NKT cell stimulation assay using NKT hybridoma cell lines and ELISA** were performed as previously described [59,97,100]. During immunofluorescence staining, to survey the CD1d protein levels upon expression of SARS-CoV-2 E protein or its transmembrane domain, one hundred transfected cells expressing E protein or its TM domain or untransfected control cells were tallied for the relative protein level of mature CD1d measured by immunofluorescence staining of mature CD1d proteins with CD1d.51.1 antibodies. Fluorescence signal levels from CD1d staining in individual cells were measured with Nikon Elements Imaging Software (Nikon Inc.). Cells with 70–100%, 20–70% or below 20% of average CD1d protein level in untransfected cells are designated as normal, partial or no CD1d protein expression.
